# Supplementary material for: Learning curve and surgical outcome of robotic assisted colorectal surgery with ERAS program
Source: Sci Rep. 2022 Nov 29;12:20566. doi: 10.1038/s41598-022-24665-w (PMC9709162; doi:10.1038/s41598-022-24665-w)
Supplement: Supplementary file 1 — Supplementary Information 1. [file 41598_2022_24665_MOESM1_ESM.zip › LSC Raw data-Ñ[▒K/091/H_ñw╝╨░O▒Kñσ.pdf]

正式摘要(電子簽章：000000198898717)

W55 / W55 ☐英文標題(注意:無英文姓名-請補病人基本檔!) ☐【讀取】憑證

臺中榮民總醫院

出院病歷摘要

轉入號： 轉出號：

[redacted]

(8)流水編號	
(9)入院日期	109/09/09 (2020) 直腸外科 W55 - 112
(10)轉科日期	
(11)出院日期	109/09/15 (2020) 住院天數計 6 日

診斷

- 入院 :Malignant tumor of sigmoid colon
- 出院 :1.Sigmoid colon cancer, s/p laparoscopic Anterior resection on 20200810, cT3N1M0
  2. HTN under medicine control

主訴

- Referred from CRS clinic ,highly suspect colon cancer

病史

- This is a 66 y/o female who has the history of
  1.HTN under medication control
  2.Spondylolisthesis s/p operation in 台中國軍H 3 years ago.
  3.Sinusitis s/p operation in 仁愛H 8 years ago.
  According to the patient , she had difficult in defecation 1 month ago.She went to clinic for help , where colonscopy showed maligancy tuumor of sigmoid colon, then she was transferred to 中榮H.Lab data showed CEA 5.93, hs-CRP 0.429 Throughout the course, there was no fever, nausea, vomiting, acute dyspnea.
  Under the impression of malignant tumor of sigmoid colon,she was admitted to our ward for further treatment

體檢發現

- Anal inspection:
  Recto digital examination:
  Sigmoidoscopy:
  Anoscopy:
  Abdomen:
  - General appearance :
    - easy looking , conscious clear , without cardiopulmonary distress
    - body weight : 48.7 kg , height : 144.3 cm
    - Vital signs自動帶入date 體溫(BT) 脈搏(P) 呼吸(R) 血壓(BP) 身高(BH) 體重(BW) BMI
      20200909 35.8℃ 73次/分 18次/分 143/84mmHg 144.3cm 48.7kg 23.4
    - Vital signs: B.P. 143/84 mmHg , P.R. 73 /min , R.R. 18 /min , B.T. 358 ℃
  - HEENT :
    - Eyes: no anemic conjunctiva, no icteric sclera, normal EOM
    - ENT: no otorrhea, no rhinorrhea, no erythematous mucosa, no injected tonsil, no deformity
  - Neck: supple, freely movable, trachea midline,no deformity, no engorged jugular vein , no palpable thyroid or lymph node enlargement
  - Chest:
    - Inspection: Grossly normal, symmetric and fully expansion, no accessory muscle used, no operation scar, no spider angiomata, no gynecomastia
    - Palpation: no palpable mass or nodule, no subcutaneous emphysema, no tenderness
    - Ausculation: clear breathing sound
  - Heart:
    - Palpation: no thrill, no heave
    - Percussion: normal heart size
    - Ausculation: regular heart beat, normal S1, S2, no gallop, no murmur
  - Extremities: No deformity, full ranged of motion without limitation, no clubbing finger or nail cyanosis, no pitting edema, no ulcer or pigmentation, no obvious skin lesion, no varicose vein, no bone, joint or muscle tenderness

  CRS CONDITION

  - Karnofsky performance status: (100) Normal, no complaints, no evidence of disease
  - PE of Abdomen:
  - CEA: 5.93 ng/ml
  - Imaging Studies:
  - Colonoscope report: maligancy tuumor of sigmoid colon
  - Figures:
    1. colon & rectum

手術日期及方法

- 手術日期及方法20200910 1. Laparoscopic anterior resection with end-to-side anastomosis, endo-GIA 60/4.1mm*1, ILS 31mm, re-inforcement with silk: nil, bilateral doughnuts: intact, air leak test: done, no air leak.
  2. vessel ligation: IMA, IMV.
  3. Cut margin: proximal 4cm, distal 7cm.
  4. Reperitonealization: nil.
  5. Take down of splenic flexure: yes.

住院治療經過

- Operation was done on 9/10. After operation, we keep ERAS protocol for her. She was under well recovery status. Under stable condition, we arranged discharge for her.

合併症

一般檢查

- 累積報告：

DATE	TIME	ALB	TP	BIL.T	BIL.D	ALKP	AST	ALT	LDH	CHOL	TG	HDL-C	LDL	UA	NH3	AMY
1090909	2027		6.7													
1090910	1520	3.7		0.35			17	12								

DATE	TIME	WBC	RBC	HGB	HCT	MCV	PLT	BLST	NEUT	BAND	SEG	LYM	MON	EOS	BAS
1090910	1520	10380		11.4			273		86.7			11.1	1.9	0.1	0.2
1090914	0944	8990	3.59	10.5			296		70.4			20.5	5.5	3.3	0.3

DATE	TIME	NA	K	CL	CA	BUN	CREAT	Mg	P	CRP	ESR	PCT	LIPASE	LACTATE
1090910	1520	140	2.2			8	0.71							
1090911	0931		3.2					1.8	2.9					
1090914	0929	136	3.5		8.2	12	0.79							

- 放射線報告：檢驗項目 **:CT ABDOMEN-SURVEY** 報告日期 **:1090910**

主　訴：
Result:
Finding:
Imaging Report Form for Colorectal Carcinoma (AJCC 8th)
1. Imaging Date: 20200909
Imaging Modality, Imaging by
CT scan
Imaging Protocol: Post IV contrast CT scan of the whole abdomen with 600cc oral contrast
2. Tumor location / Size
Location:
Sigmoid
Size:
Non-measurable
3. Tumor invasion
Tumor within colonic or rectal wall
4. Regional nodal metastasis
Not assessable
5. Distant metastasis (In this study)
No or Equivocal
6. Other findings: > Moderate wall-thickening over the junction of D-colon and S-colon(axial:48), colon Ca. may be compatible.
> Three small LNs are next to the thickened S-colon(axial:48, 49), nature to be determined.
> A 7.2mm nodule arising from gallbladder fundus, R/O polyp.
> DJD change of T-L spine with spur formation.
> S/P internal fixation of L5-S1 with strong beam-hardening.
artifact, which may conceal subtle lesions.
Impression:
1. Compatible with S-colon Ca. Three small LNs are next to the thickened S-colon(axial:48, 49), nature to be determined. Imaging stage T1-2NxM0.
2. R/O gallbladder polyp.

特殊檢查

病理報告

其它

出院時情況

- 3.改門診治療

出院指示

藥名	劑量	劑量單位	用法	頻次	數量	數量單位	現況	天數	單位
(I I)Takepron OD tab 30mg	1	TAB	PO	QDAC	14	TAB	TH	14	天
Mag. Oxide tab	2	TAB	PO	TID	84	TAB	TH	14	天
Through tab	1	TAB	PO	QNPRN	14	TAB	TH	14	天
TramTOR cap 50mg	1	CAP	PO	QID	28	CAP	TH	7	天
Lactam tab 500mg	1	TAB	PO	Q6HPRN	20	TAB	TH	5	天

出院計劃

主治醫師 2062F 蔣鋒帆 / 住院醫師 2330E 楊捷儒

正式摘要(電子簽章：**000000198898717)**

W55 / W55

doctorRank:B
